# medPJplus – development and implementation of a concept for the acquisition and qualification of teaching practices for the final year in family medicine at the University Medical Center Göttingen

**DOI:** 10.3205/zma001434

**Published:** 2021-02-15

**Authors:** Iris Demmer, S. Borgmann, E. Kleinert, A. Lohne, E. Hummers, F. Schlegelmilch

**Affiliations:** 1 University Medical Center Göttingen, Department of General Practice, Göttingen, Germany

**Keywords:** undergraduate medical education, final year, family medicine, teaching practice, preceptor recruitment, rural area

## Abstract

**Aim: **The Masterplan Medizinstudium 2020 (Masterplan for Medical Studies 2020) focuses on practice-oriented undergraduate training with increased involvement of rural teaching practices. The demand for teaching practices for the final year will increase at all medical faculties in Germany. The project medPJplus at the University Medical Center Göttingen (UMG) presents an approach for successfully acquiring general medical teaching practices in local rural areas.

**Project outline: **The project medPJplus implemented eight measures in cooperation with medical students, interested general practitioners, and regional players in the surrounding districts to attract new teaching practices: we established public relations, accredited practices, organized the didactic training of participating general practitioners, created a digital platform for students that is linked to the nationwide PJ-Portal, and organized information events, workshops, and feedback reports to regional actors.

**Results: **Within one year, a total of 40 new teaching practices with 57 new teachers in the local rural area joined the project in southern Lower Saxony. A three-stage didactic training concept for general practitioners was established at the UMG. A digital platform enhances the visibility of general practitioners and their activities for students. The teaching practices can now be found on the nationwide PJ-Portal. Fourteen students have currently completed their period of the final year in family medicine there.

**Conclusions: **It is possible to acquire rural general medical teaching practices for the final year. This depends on four core elements: addressing and didactic training of interested general practitioners, networking of medical students with teaching physicians and regional actors, digitally presenting teaching practices, and developing solutions for mobility and living space during the final year.

## Zusammenfassung

**Zielsetzung: **Der Masterplan Medizinstudium 2020 fordert für Medizinstudierende eine praxisnahe ärztliche Ausbildung mit vermehrtem Einbezug ländlich gelegener Lehrpraxen. Der Bedarf an Lehrpraxen für das Praktische Jahr (PJ) wird an allen medizinischen Fakultäten in Deutschland steigen. Das Projekt medPJplus zeigt das Vorgehen für die erfolgreiche Gewinnung allgemeinmedizinischer PJ-Lehrpraxen in ländlicher Umgebung der Universitätsmedizin Göttingen (UMG) auf.

**Projektbeschreibung: **Im Projekt medPJplus wurden acht Maßnahmen zur Gewinnung neuer PJ-Lehrpraxen in Kooperation mit Medizinstudierenden, interessierten Hausärzten und regionalen Akteuren der umliegenden Landkreise durchgeführt: Öffentlichkeitsarbeit, Praxenakkreditierung, didaktische Fortbildung teilnehmender Hausärzte, Aufbau einer digitalen Plattform für Studierende, Verlinkung zum bundesweiten PJ-Portal, GetTogether- und Informationsveranstaltungen, Workshops und Feedbackberichte an regionale Akteure.

**Ergebnisse:** Innerhalb eines Jahres wurden in Südniedersachsen insgesamt 40 neue PJ-Lehrpraxen mit 57 neuen PJ-Lehrärzten im ländlichen Umfeld der UMG gewonnen. Ein dreistufiges didaktisches Fortbildungskonzept für Hausärzte wurde an der UMG etabliert. Eine digitale Plattform macht allgemeinmedizinische Lehrärzte und ihre Tätigkeit für Studierende sichtbarer. Die Lehrpraxen sind jetzt im bundesweiten PJ-Portal auffindbar. 14 Studierende absolvierten dort bereits ihr allgemeinmedizinisches PJ.

**Schlussfolgerungen: **Die Gewinnung ländlich gelegener allgemeinmedizinischer Lehrpraxen für das Praktische Jahr ist möglich. Sie hängt von vier Kernelementen ab: Ansprache und didaktische Fortbildung interessierter Hausärzte, Vernetzung von Medizinstudierenden mit Lehrärzten und regionalen Akteuren, Digitalisierung der PJ-Suche sowie Erarbeitung von Lösungen für Mobilität und Wohnraum während des PJ.

## 1. Introduction

With the Masterplan for Medical Studies 2020, the Federal Ministry of Health addresses a major challenge for the health care system in Germany: “comprehensive general medical care in rural and structurally weak regions with existing or emerging shortage of medical care” [1]. This is associated with a restructuring of medical studies and the medical license for practicing medicine. The focus of the Masterplan lies on practice-oriented training and the increased integration of teaching practices into medical training [2]. The recruitment of graduates of medical studies for family practice in rural areas is considered a task of public interest. A commission of experts has proposed the longitudinal structure of general practice courses as a concrete measure [1]. 

Innovations are planned for medical training in the general practice for clinical elective, the so-called Blockpraktikum (a two-week internship in a general practice in the fifth year at university), the last phase of undergraduate medical education, the final year (Praktisches Jahr, PJ), and licensing examinations (see table 1 (Tab. 1)). It is assumed that the introduction of a compulsory outpatient quarter will increase the proportion of PJ students in general medicine to about one-third of the students in a year group [3]. This quota might even be exceeded: according to the draft of the new Licensing Regulations for Doctors (Ärztliche Approbationsordnung, ÄApprO), a patient from general outpatient care is to be treated for the examination in the planned Fourth Section of the Medical Examination (M4), regardless of the choice of the compulsory outpatient subject. In addition, general medical wards are provided for the structured clinical-practical examination (§30, paragraph 4 and §15, paragraph 9 of the draft of the new ÄApprO) [4]. In order to meet these planned innovations, the medical faculties need a significantly higher number of general medical teaching practices and examiners. 

For the University Medical Center Göttingen (UMG), where about 360 students per year study medicine, this means that about 120 or more students per year will complete the compulsory outpatient subject in a general medical teaching practice in the future [5], [6]. The structured approach to the acquisition of general medical practices has so far been reported for research projects, but not for teaching purposes [7]. Assuming that two to three PJ students per year can be supervised in a practice, this requires about 40 to 60 PJ teaching practices. 

All medical faculties in Germany must meet the challenge of integrating a number of general medical practices and medical care centers (Medizinische Versorgungszentren, MVZ) especially in rural areas in the vicinity of the universities, into medical training in block internships and PJs. This is required to ensure the quality of decentralised teaching in all teaching practices and to recruit the teaching physicians as examiners for the Fourth Section of the Medical Examination. 

In particular, the PJ contributes to setting the course with regard to the choice of specialisation in continuing medical education [8], [9]. The creation of rural training locations for medical students in the vicinity of their universities is intended to increase the chances that the graduates will continue their further medical training in this region after completing their studies and later become (general) physicians there. At the same time, this is in the interest of the regions close to the locations of the university.

The medPJplus project, which is short for Medical Practical Year in a Family Practice in Southern Lower Saxony, addresses the care-specific interests of the region of southern Lower Saxony [10], [11], where the University Medical Center Göttingen is located, as well as university challenges for the implementation of the Master Plan for Medical Studies 2020. The project develops concrete solutions. It was funded from 27 November 2018 to 31 December 2019 by the Ministry of Federal and European Affairs and Regional Development of Lower Saxony “Regional Development Impulses in Lower Saxony” through the offices for regional state development Braunschweig and Leine-Weser. The UMG also participated financially in the project with their own funds, as did the districts of Goslar, Göttingen, Holzminden, and Northeim. 

The interprofessional project team (the authors) of the Department of General Practice consisted of a medical project manager, a pedagogue, a computer scientist, a social scientist, and an assistant. 

Important cooperation partners were general practitioners (GP) in private practice and regional actors from the districts of Goslar, Göttingen, Holzminden, and Northeim. Furthermore, the medPJplus project was included in the Southern Lower Saxony Program [https://www.suedniedersachsenprogramm.niedersachsen.de/startseite/] and was conducted in cooperation with the Göttingen health region and the Göttingen district office of the Association of Statutory Health Insurance Physicians of Lower Saxony.

The aim of this project report is to present the approach, describe the concrete measures we implemented to attract new PJ teaching practices in southern Lower Saxony, and report the results of the project. Core elements for the successful acquisition of rural PJ teaching practices were identified and offer suggestions for other medical faculties on how to proceed in the local region of their university. 

## 2. Project description

### 2.1. Initial situation

In order to implement the compulsory outpatient quarter that is planned in the Masterplan for Medical Studies 2020, the Faculty of Medicine of the UMG requires significantly more general medical PJ teaching practices than the 23 sites accredited to date. Based on the assumption that 30% to 60% of the 360 students per year complete their compulsory outpatient quarter in a general medical PJ teaching practice, 120 to 240 PJ places per year are required in the medium term. There is great interest in being able to provide these places in southern Lower Saxony [10], [12]. At the same time, a high quality of decentralised PJ training in the practices must be ensured. This includes both the equipment and the range of treatments, and also the didactic qualifications of the general practitioners involved in PJ training. Furthermore, the procedure for registering UMG students for the outpatient PJ section is to be digitised. This will be implemented via the PJ portal, which is widely used in Germany.

In each of the four administrative districts, open information events were held for interested general practitioners and regional players in the districts and municipalities. Participating general practitioners were highly motivated to train for PJ teaching practices in cooperation with the UMG and to teach students the general medical spectrum of activities and the corresponding skills and abilities. They were interested in a personal exchange with students and in making their practices visible to students on a digital platform. The regional players expressed a high level of interest in cooperating with the UMG and declared their willingness to provide impulses for student-support possibilities with regard to mobility and housing and to make their regions more attractive to students. In addition, the regional actors hoped that the project would provide feedback from medical students on their region as a place to work and live, and in the medium term, to attract young GPs, especially to the rural areas of southern Lower Saxony.

In individual interviews, requests for the project were recorded by students of the UMG, general practitioners in private practice, and regional actors (county employees, including health authorities, mayors, and members of citizens' initiatives) of the counties involved in the project. The project objectives that were derived are described below.

#### Project goals for students

Students of the UMG are provided with 40 additional general medical practices for a PJ in the four districts of Goslar, Göttingen, Holzminden, and Northeim. A digital platform informs them about the PJ teaching practices as well as the respective location. Current events of the Department of General Practice for students on the general medical PJ are listed there as well. The students are given the opportunity to personally exchange information with PJ teaching physicians and regional stakeholders. The PJ teaching practice can be booked via the PJ portal.

##### Project goals for teaching physicians 

General practitioners in private practice train as PJ teachers within the framework of a structured didactic programme. Their practices are accredited as PJ teaching practices by the Department of General Practice of the UMG. They exchange information with students of the UMG. In the long term, the general practitioners actively contribute to the recruitment of young family doctors in southern Lower Saxony by training PJ students in their practices.

##### Project goals for regional players

Regional actors develop strategies to increase the attractivity of their region as a location for general medical PJ teaching practices in exchange with medical students and the UMG project team. They receive feedback on the needs and expectations of medical students regarding their region as a potential working and living space. In the long term, regional actors benefit from the fact that students develop a motivation to complete their medical training in this region and to stay there later and become general practitioners. 

Concrete project measures were defined to achieve the project objectives (see table 2 (Tab. 2)).

#### 2.2. Implementation of the project 

The project was implemented in four phases: preparation (12/2018), implementation I (01/2019 to 04/2019) and II (05/2019 to 11/2019), and evaluation (12/2019). The preparation phase served for team building, networking with regional actors, and planning all events for 2019. In the first implementation phase, the focus was on gaining new teaching practices. The second implementation phase focused on the further training of GPs; the personal interaction between students, GPs, and regional actors; and digital networking on the project homepage. During the evaluation phase, feedback reports were prepared for the districts. 

In order to obtain general medical teaching practices, we were guided by the postcode areas for the districts of Goslar, Göttingen, Holzminden, and Northeim [https://www.suche-postleitzahl.org/]. These are the neighbouring areas of the UMG. We also used the portal “Arztsuche Niedersachsen” (Search for Doctors in Lower Saxony) to search for general practitioners and general internists in private practice [https://www.arztauskunft-niedersachsen.de/ases-kvn/], informed all doctors who could be found by post with a project profile, and invited them to participate. In addition, the regional players involved addressed their local GPs personally. Interest in participating was also sought from the teaching physicians for block internships and PJs that have cooperated with the UMG before, as well as from regional medical associations. We followed the recommendations of the Association of University Teachers and Lecturers in General Medicine e.V. [13] when we defined the general requirements for general medical PJ teaching practices and the accreditation criteria at the beginning of the course and continuous teaching in the practice. For the medical didactic training of general practitioners, we designed a free three-stage compulsory programme with a beginner's course, advanced courses I and II, and a voluntary qualification as a medical examiner for the current final medical exam. 

The development of the digital platform [https://www.medpjplus.de/] was divided into three phases (basic structure and layout, filling with content, testing of the legally compliant appearance) over the project duration. For the personal interaction of medical students with general practitioners and regional actors, we organised get-together events. 

The topic “accessibility of medical practices for PJ students in rural areas” was addressed in a workshop "mobility and housing” with regional actors. First ideas for housing and mobility were collected and discussed during the PJ in two small groups.

## 3. Results

In the following we describe the results we obtained for project measures M1 to M8 (see table 2 (Tab. 2)). 

### M1: Obtaining general medical teaching practices

Before the start of the medPJplus project (i.e. before 2019), the PJ teaching practices that collaborated with the UMG were concentrated mainly in the area of Göttingen itself. In addition, PJ teaching practices participated in four rural locations in southern Lower Saxony, and in isolated locations in other areas of Lower Saxony and neighbouring federal states.

**Figure 1:**
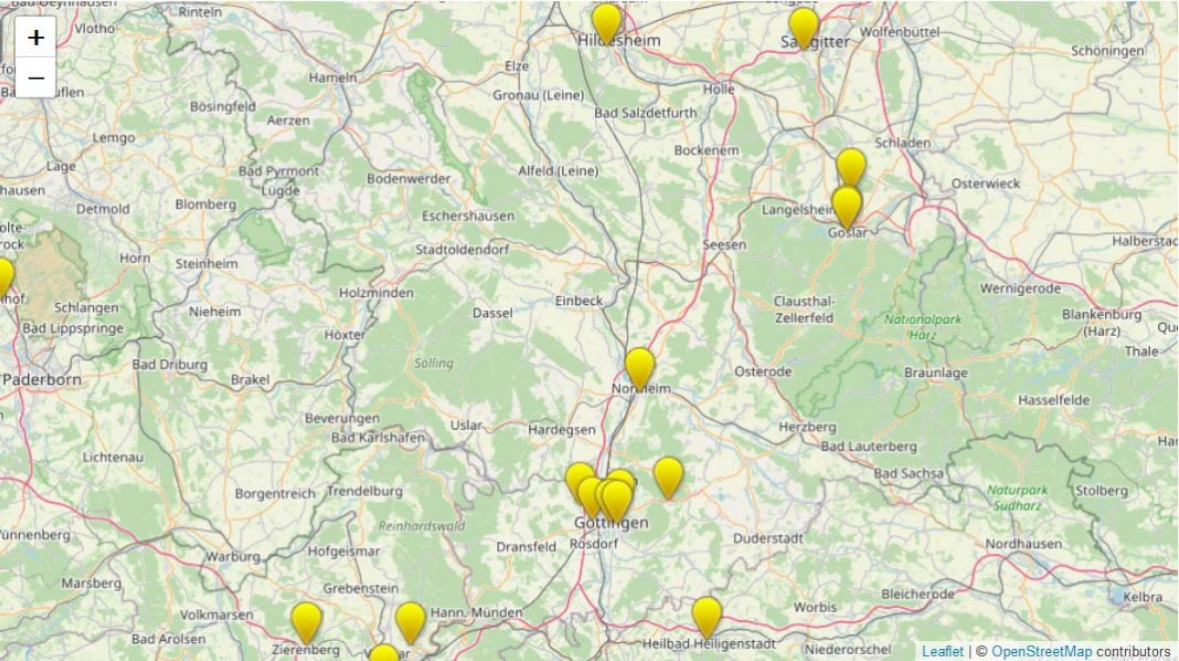
Locations of the PJ teaching practices of the Department of General Practice of the University Medical Center Göttingen before the start of the project in December 2018.

**Figure 2:**
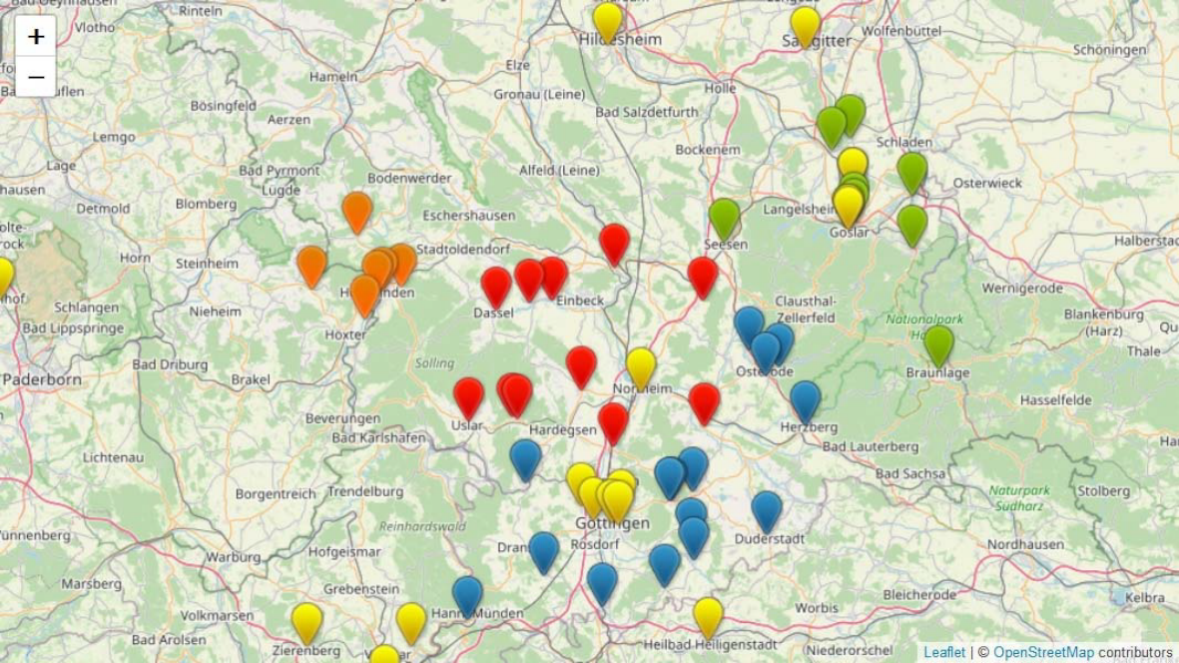
Locations of the PJ teaching practices of the Department of General Practice of the University Medical Center Göttingen at the end of the project (31 December 2019). The new PJ teaching practices in the districts of Goslar (green), Göttingen (blue), Holzminden (orange) and Northeim (red) are marked.

A total of 38 new general medical teaching practices and two MVZs in southern Lower Saxony with a total of 57 cooperating general practitioners were recruited within one year (see table 3 (Tab. 3)) as a result of the medPJplus project. A comparison of the geographical location data of the PJ teaching practices before 2019 (see figure 1 (Fig. 1)) and after 31 December 2019 (see figure 2 (Fig. 2)) illustrates the increase in the density of the teaching practice network in the area of southern Lower Saxony. The comparison also shows that many of the newly acquired practices are located in rural areas.

#### M2: Didactic training programme

A didactic training programme for PJ teachers was developed. This includes the subject areas (organisational matters, teaching content, learning objectives, and feedback) desired by participating teachers and can be completed in stages in afternoon courses. It was divided into a beginners' course and an advanced course I and II (each with a three-hour classroom session), and it was evaluated. In the medium term, it is planned that PJ teachers will have completed at least the beginners’ course before they start training PJ students. In addition, each PJ teacher should have completed all courses within three years. Because of the high demand, the beginners' course was offered twice in 2019 (25 and 24 participants, respectively). In 2019, 27 and 22 PJ teachers took part in the two advanced courses, respectively. The contents of the individual courses are summarised in table 4 (Tab. 4).

#### M3/M4: Digital platform and entries in the nationwide PJ portal

The digital platform www.medpjplus.de does not only present a map of PJ teaching practices in rural areas, but also shows 19 medical practices with pictures and information. In a structured presentation, students can obtain a first impression of their PJ teaching practice and find relevant contact details of the practice, contact persons, as well as information about the team, the practice equipment, and treatment focus. The presentation on the internet makes the family doctor's work visible to students of other faculties on a national level. In cooperation with the Dean of Studies of the UMG, it was also possible to include general practitioners in private practice in the nationwide PJ portal. 

#### M5: Get-together events

The first get-together event in the summer of 2019 was attended by 35 people (15 students, 13 GPs, and 7 regional players) and the second by 14 people (8 students, 4 GPs, and 2 regional players). Overall, the events were perceived as rewarding. It was important for students to realise that the participating general practitioners are highly motivated to offer the PJ study section in their practice. Students emphasised the importance of becoming personally acquainted with the teaching physicians in order to decide on the elective subject of general practice and the appropriate practice. At the end of the events, important topics were summarised by students, general practitioners, and regional players and were then presented to all. An overview of this is given in table 5 (Tab. 5).

#### M6: Information events on PJ for students

Within the framework of the PJ fairs held each semester at the UMG, interested students were introduced to the subject of general medicine and the medPJplus project. In addition, students in the fifth clinical semester were informed about the project in the General Medicine module. With the display of flyers and invitations to the get-together events, students from other semesters also received information about the project and the opportunity to participate.

#### M7: Workshop mobility and living space

The workshop was attended by 14 regional actors from the four districts. They discussed the topics on which the regions would like to receive information from students. In two group-work sessions, two pools of ideas for offers on mobility and housing for students in the PJ in southern Lower Saxony were created, which can be developed and used as required.

#### M8: Feedback reports

The feedback reports contain all the results of project events, information about new teaching practices, public relations work, and feedback from students with questions about and requests to the regions. These reports were made available to the districts and the funding bodies.

In the course of the project, four core elements for the successful acquisition of PJ teaching practices emerged. We list them below in order of importance.

- *General practices as an academic field of training:* The attractivity and learning interest of students in practical study phases such as PJ decisively depend on the motivation and teaching quality of the teachers [14], [15]. The didactic continuing education concept from medPJplus, which will continue after the end of the project, can contribute to the quality assurance of decentralised PJ teaching in the outpatient sector required by the medical faculty.
- The *networking between* university contact persons, teaching physicians, and medical students, as well as the social recognition of the teaching profession, are motivating factors for qualification and many years of teaching activity. It enhances the willingness to provide the additional time and organisational effort for the care of medical students [16], [17]. Regional actors from southern Lower Saxony have recognised in the project that the attractivity of the surroundings has great influence on the choice of PJ location for medical students [18]. The regions can influence this in the areas of networking, mobility, housing, and the attractivity of the surroundings.
- *Digitisation of the PJ search:* Medical students can find positions for their PJ training in university clinics and academic teaching hospitals throughout Germany via a central Internet portal [https://www.pj-portal.de/]. The additional presence of general medical PJ teaching practices in the digital PJ portal increases the chances that they are selected as PJ learning locations nationwide.
- *Mobility and housing: *Integrating rural teaching practices into the academic training field of a university meets the challenges of “mobility” and “living space”, especially in structurally poor areas. Here, new solution models for on-site mobility and temporary living (for a few months in the PJ of medical students) can be developed and implemented in cooperation with the medical faculties, the teaching practices, and regional actors.

## 4. Discussion

In the medPJplus project, the Department of General Practice at the University Medical Center Göttingen succeeded in recruiting and qualifying 40 rural general medical teaching practices with 57 teaching physicians for the PJ. 

Comparable initiatives have been taken throughout Germany to increase the attractivity of general medical internships in medical studies (e.g. Aktion Landarzt Leben Lieben [https://www.perspektive-hausarzt-bw.de/aktionen/lall/], Beste Landpartie Allgemeinmedizin [https://www.kvb.de/nachwuchs/studium/beste-landpartie-allgemeinmedizin/], Landpartie 2.0 [19]). However, there are no comparable publications on the recruitment and university integration of further PJ teaching practices with regard to the implementation of the Master Plan for Medical Studies 2020. 

The medPJplus project focuses not only on the recruitment and further training of future PJ teachers, but also on the early bonding of medical students to regional players and general practitioners in private practice in southern Lower Saxony. The effort and expense involved is worthwhile because approximately 49.3% of the students are interested in working as general practitioners [20], [21] and because the planned new medical licence for practising medicine in the next few years will make a compulsory quarter of outpatient training in general medical practices possible. 

Limiting factors of the project were the regional framework, the necessary third-party funding for scientific personnel (0.75 full-time equivalent, FTE) and assistants (0.5 FTE), and the short project duration (12 months). However, the procedure developed in the project for gaining practical experience, the continuing education programme, and the digital platform were continued after the end of the project without further third-party funding. In this way, we have been able to acquire additional general medical PJ teaching practices since the beginning of 2020, to run the continuing education programme as online courses even under pandemic conditions, and to place 14 PJ students in general medicine as an optional subject in practices in southern Lower Saxony. The continuation of get-together events is desirable. Students can use personal contacts with PJ lecturers in these events to reach a decision for their PJ. The concepts and results from this project can also be helpful in other medical faculties and help them gain general medical PJ teaching practices, taking into account regional conditions. Our results contribute to the availability of general medicine in the future, not only as a PJ elective, but also for the compulsory outpatient quarter that is planned with the new medical licensing regulations. University cooperation with all PJ teaching practices and the quality of their PJ teaching should be evaluated regularly. 

## 5. Conclusions

It is possible to acquire rural general medical practices as teaching locations for the PJ. If the associated challenges and the necessary cultivation of relationships between the medical faculty, general practitioners in private practice, and regional players are approached as joint tasks and personal contact between students and PJ lecturers is promoted, the initially high effort can subsequently lead to benefits for all parties involved and strengthen the subject of general medicine. 

## Funding

The medPJplus project was funded with 84.000 € from the funding programme “Regional Development Impulses in Lower Saxony” in the period from 19 November 2018 to 31 December 2019 and co-financed by the districts of Goslar, Göttingen, Holzminden, and Northeim with a total of 16.000 €. The Medical Faculty of the University of Göttingen also contributed 12.000 €. 

## Competing interests

The authors declare that they have no competing interests. 

## Figures and Tables

**Table 1:**
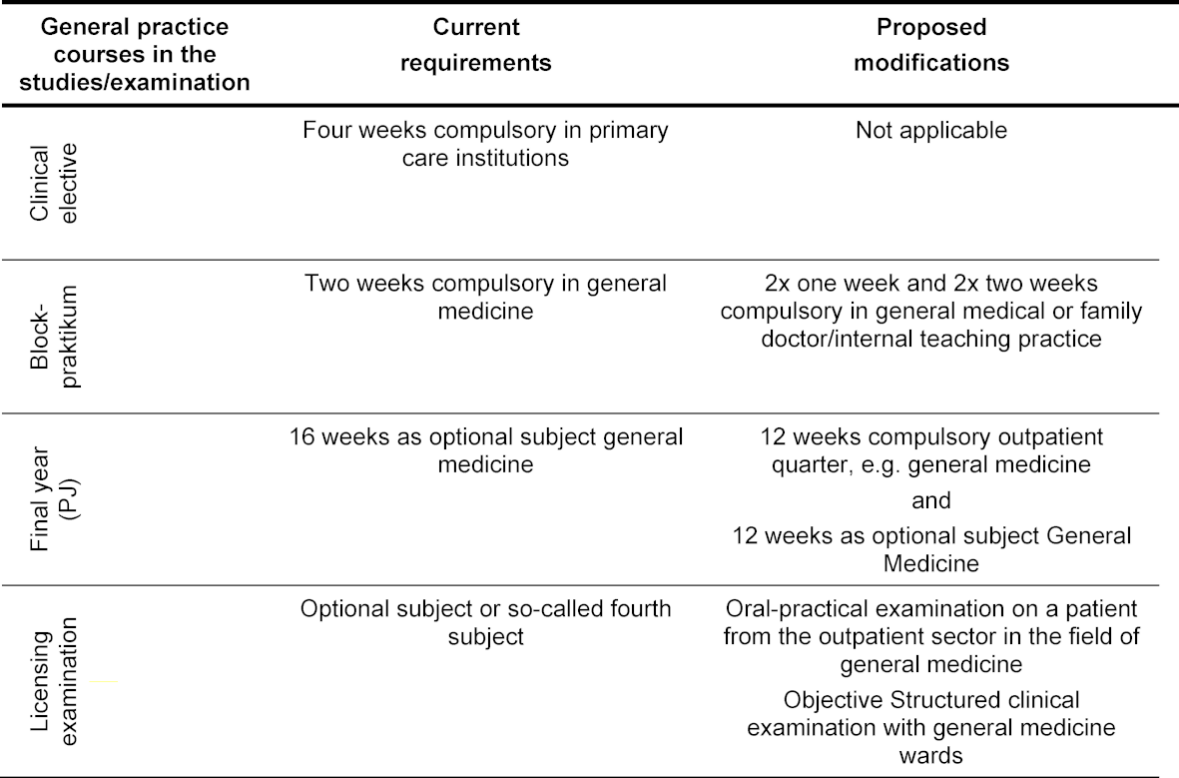
General practice study sections and last section of the medical examination in the currently valid Licensing Regulations for Doctors [https://www.gesetze-im-internet.de/_appro_2002/BJNR240500002.html] and planned changes through the working draft of the new Licensing Regulations for Doctors [4].

**Table 2:**
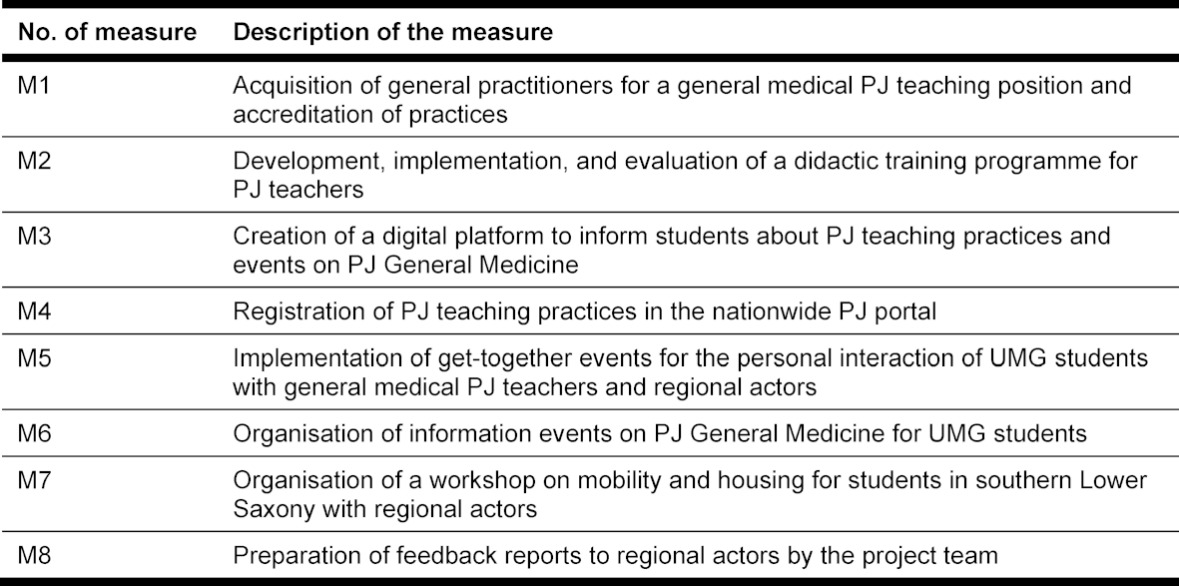
Overview of the project measures

**Table 3:**
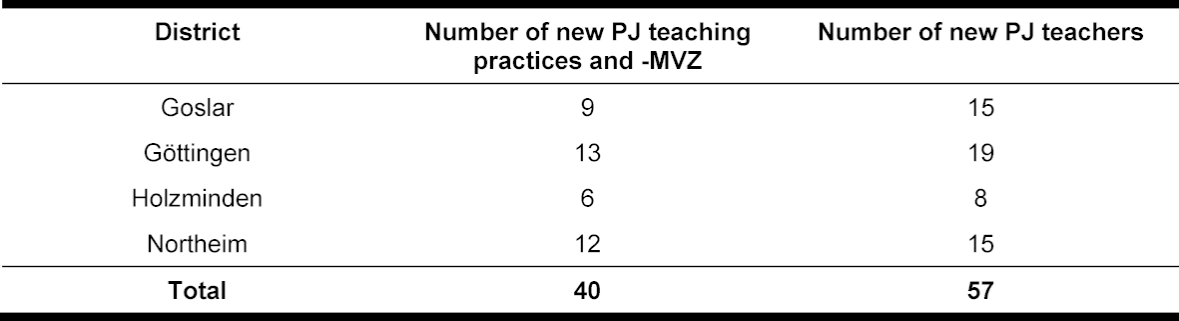
Number of new PJ teaching practices and PJ teachers acquired through the medPJplus project by district

**Table 4:**
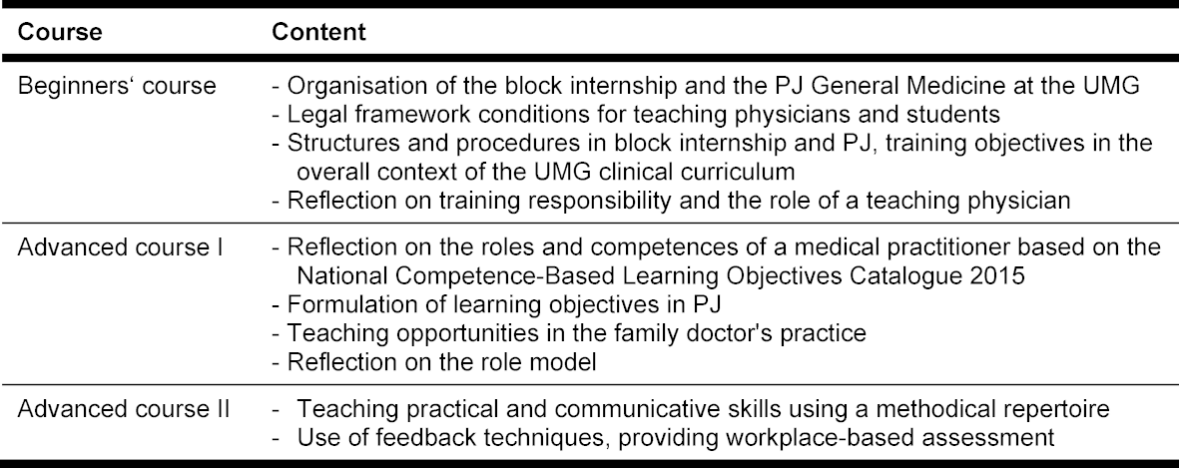
Content of the individual courses of the didactic training concept

**Table 5:**
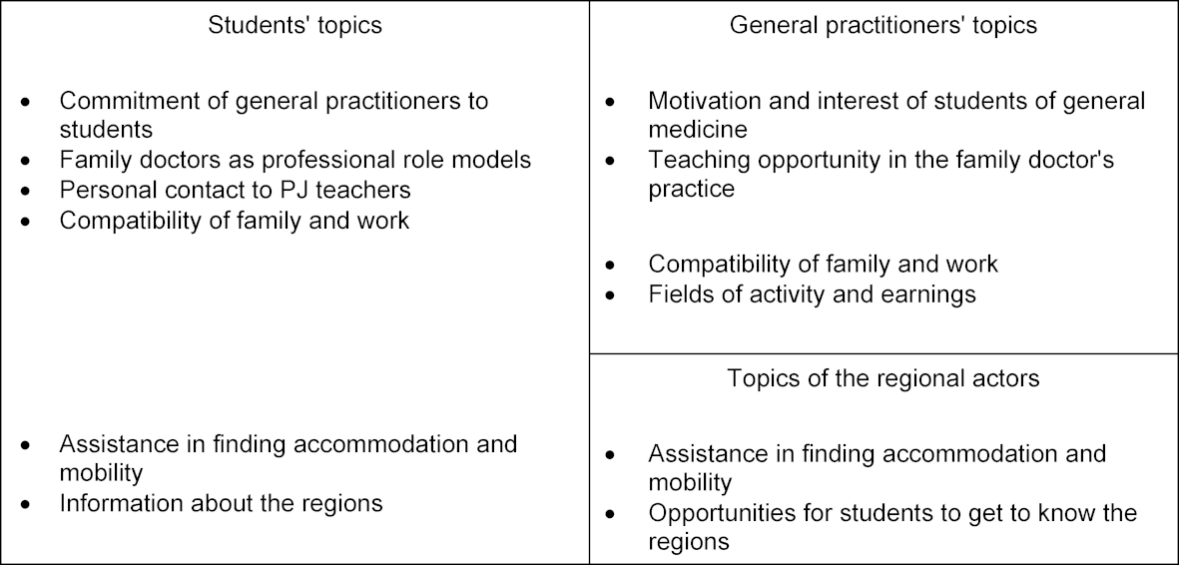
Comparison of topics during the get-together events

## References

[1] Wissenschaftsrat (2018). Neustrukturierung des Medizinstudiums und Änderung der Approbationsordnung für Ärzte.

[2] Bundesministerium für Bildung und Forschung (2017). Masterplan 2020.

[3] Petruschke I, Schulz S, Kaufmann M, Bleidorn J (2019). Masterplan Medizinstudium 2020 - für welches ambulante Wahlquartal entscheiden sich Studierende?.

[4] Bundesärztekammer (2020). Synopse Approbationsordnung für Ärzte (ÄApprO) aktuelle Fassung - Arbeitsentwurf.

[5] DEGAM (2013). Pflichtquartal Allgemeinmedizin im Praktischen Jahr.

[6] Kötter J (2020). Neues Medizinstudium: Praxisbezug ab Tag eins. Z Allg Med.

[7] Güthlin C, Beyer M, Erler A, Gensichen J, Hoffmann B, Mergenthal K, Müller V, Muth C, Petersen JJ, Gerlach FM (2012). Rekrutierung von Hausarztpraxen für Forschungsprojekte - Erfahrungen aus fünf allgemeinmedizinischen Studien. Z Allg Med.

[8] Böhme K, Siegel A, Kotterer A, Streitlein-Böhme I, Maun A (2018). PJ-Wahlfach Allgemeinmedizin - eine Weichenstellung für die Hausarztkarriere?. Z Allg Med.

[9] Böhme K, Kotterer A, Simmenroth-Nayda A (2013). Allgemeinmedizin im Praktischen Jahr - eine Lösung für Nachwuchsprobleme in der hausärztlichen Versorgung? Ergebnisse einer multizentrischen PJ-Evaluation. Z Allg Med.

[10] Amt für regionale Landesentwicklung Braunschweig (2018). Regionale Handlungsstrategie.

[11] Amt für regionale Landesentwciklung Leine-Weser (2015). Hausarzt (m/w/d) gesucht!.

[12] Amt für regionale Landesentwicklung Leine-Weser (2017). Broschüre Reginale Handlungsstrategie Leine-Weser.

[13] Wilm S, Klinsing U, Donner-Banzhoff N (2003). Allgemeinmedizinische Lehrbeauftragte, Lehrärzte und akademische Lehrpraxen.

[14] Mahler D, Großschedl J, Harms U (2018). Does motivation matter? - The relationship between teachers' self-efficacy and enthusiasm and students' performance. PloS One.

[15] Dybowski C, Sehner S, Harendza S (2017). Influence of motivation, self-efficacy and situational factors on the teaching quality of clinical educators. BMC Med Educ.

[16] Deutsch T, Winter M, Lippmann S, Geier A-K, Braun K, Frese T (2019). Willingness, concerns, incentives and acceptable remuneration regarding an involvement in teaching undergraduates - a cross-sectional questionnaire survey among German GPs. BMC Med Educ.

[17] Klement A, Ömler M, Baust T, Bretschneider K, Lichte T (2011). Lehrmotivation und Evaluationsbereitschaft - eine explorative Querschnittsstudie unter Lehrärzten: Motivation for teaching and evaluation - an explorative cross sectional study among FP-preceptors. Z Allgemeinmed.

[18] Homberg A, Narciß E, Schüttpelz-Brauns K (2019). What reasons do final-year medical students give for choosing the hospitals for their clinical training phases? A quantitative content analysis. GMS J Med Educ.

[19] Seeger L, Becker N, Ravens-Taeuber G, Sennekamp M, Gerlach FM (2020). "Landpartie 2.0" - Conceptual development and implementation of a longitudinal priority program to promote family medicine in rural areas. GMS J Med Educ.

[20] Bien A, Ravens-Taeuber G, Stefanescu MC, Gerlach FM, Güthlin C (2019). What influence do courses at medical school and personal experience have on interest in practicing family medicine? - Results of a student survey in Hessia. GMS J Med Educ.

[21] Jacob R, Kopp J, Schultz S (2014). Berufsomitoring Medizinstudenten 2014. Ergebnisse einer bundesweiten Befragung.

